# Dimension Reduction and Clustering Models for Single-Cell RNA Sequencing Data: A Comparative Study

**DOI:** 10.3390/ijms21062181

**Published:** 2020-03-22

**Authors:** Chao Feng, Shufen Liu, Hao Zhang, Renchu Guan, Dan Li, Fengfeng Zhou, Yanchun Liang, Xiaoyue Feng

**Affiliations:** 1 Key Laboratory of Symbolic Computation and Knowledge Engineering of the Ministry of Education, College of Computer Science and Technology, Jilin University, Changchun 130012, Chinaliusf@jlu.edu.cn (S.L.); haozhang17@mails.jlu.edu.cn (H.Z.); guanrenchu@jlu.edu.cn (R.G.); ffzhou@jlu.edu.cn (F.Z.); ycliang@jlu.edu.cn (Y.L.); 2 Zhuhai Sub Laboratory of Key Laboratory of Symbolic Computation and Knowledge Engineering of the Ministry of Education, Zhuhai College of Jilin University, Zhuhai 519041, China; 3 Joint Bioinformatics Program, University of Arkansas Little Rock George Washington Donaghey College of Engineering & IT and University of Arkansas for Medical Sciences, Little Rock, AR 72204, USA; dxli@ualr.edu

**Keywords:** single-cell RNA sequencing, dimensionality reduction, clustering algorithm

## Abstract

With recent advances in single-cell RNA sequencing, enormous transcriptome datasets have been generated. These datasets have furthered our understanding of cellular heterogeneity and its underlying mechanisms in homogeneous populations. Single-cell RNA sequencing (scRNA-seq) data clustering can group cells belonging to the same cell type based on patterns embedded in gene expression. However, scRNA-seq data are high-dimensional, noisy, and sparse, owing to the limitation of existing scRNA-seq technologies. Traditional clustering methods are not effective and efficient for high-dimensional and sparse matrix computations. Therefore, several dimension reduction methods have been introduced. To validate a reliable and standard research routine, we conducted a comprehensive review and evaluation of four classical dimension reduction methods and five clustering models. Four experiments were progressively performed on two large scRNA-seq datasets using 20 models. Results showed that the feature selection method contributed positively to high-dimensional and sparse scRNA-seq data. Moreover, feature-extraction methods were able to promote clustering performance, although this was not eternally immutable. Independent component analysis (ICA) performed well in those small compressed feature spaces, whereas principal component analysis was steadier than all the other feature-extraction methods. In addition, ICA was not ideal for fuzzy C-means clustering in scRNA-seq data analysis. K-means clustering was combined with feature-extraction methods to achieve good results.

## 1. Introduction

Owing to the development of microfluidics, large numbers of cells can now be isolated [1]. Advances in RNA isolation and amplification have resulted in the application of RNA-sequencing (RNA-seq) technology to analyze the transcriptomes of single cells [2,3,4]. The technology has spurred the creation of several atlas projects, such as the Human Cell Atlas [5]. In 2017, 10X Genomics released a dataset of 1.3 million mouse brain cells, which was the largest dataset published in the single cell RNA-sequencing (scRNA-seq) field. Large-scale single-cell data provide new methods to address biological problems; however, they pose specific analytical and technical challenges, such as high dimensionality, sparse matrix computation, and rare cell type detection [6,7]. A high-dimensional and sparse matrix will cause the curse of dimensionality. Therefore, the computational analysis of scRNA-seq data involves several steps, including quality control, mapping, quantification, dimensionality reduction, clustering, finding trajectories, and identifying differentially expressed genes [4]. Among these techniques, dimensionality reduction and clustering are two of the most important steps that have substantial effects on downstream analysis.

As a large number of genes are assayed in single-cell RNA-seq data, distances between samples (i.e., cells, in our case) tend to be small and not reliable for cluster identification (i.e., cell groups, in our case) in this high-dimensional feature space [4]. This challenge is known as the curse of dimensionality. To alleviate this problem and other undesirable properties of high-dimensional space, dimensionality reduction models have been applied in many fields [8,9]. To obtain a data-driven, coherent, and unbiased approach and to discover the natural groupings of a set of samples, we focused on unsupervised dimensionality reduction methods.

For scRNA-seq transcriptome data, unsupervised clustering models have been used in several studies to define new cell types. For example, the K-means clustering algorithm is one of the top 10 most widely used data mining algorithms [10], and it has been used in the Monocle scRNA-seq toolkit [11]. Hierarchical clustering is another widely used clustering algorithm in scRNA-seq data analysis, which combines each sample (a single cell, in our case) into larger clusters sequentially or divides large clusters into smaller groups. Some scRNA-seq tools, such as BackSPIN [12] and pcaReduce [13], extend hierarchical clustering by reducing the dimensions after each split or merge. This iterative strategy improves the ability to identify small clusters. Community detection in a complex network is a variant of the clustering concept [14,15,16]. Blondel et al. [17] tested the high accuracy of the Louvain algorithm on ad hoc modular networks and demonstrated its excellent performance in comparison with other community detection methods. The toolkit of single-cell analysis in Python (SCANPY) adopted the Louvain clustering algorithm for gene expression data analysis of single cells [18]. However, comprehensive studies to determine whether clustering, dimensionality reduction, or the hybrid model is the most appropriate approach are lacking.

To achieve satisfactory results for scRNA-seq data, considerable challenges, such as data scale, technical noise, and sparsity, must be overcome. In this study, we surveyed dimensionality reduction algorithms and clustering models including filter-based feature selection (FBFS), principal component analysis (PCA), independent component analysis (ICA), non-negative matrix factorization (NMF), K-means, hierarchical clustering, Louvain, fuzzy C-means, and density-based spatial clustering of applications with noise (DBSCAN). Experiments on two benchmark scRNA-seq datasets comprehensively illustrated the effectiveness of different combinations of these dimensionality reduction and clustering models.

## 2. Results

### 2.1. Datasets

The first scRNA-seq dataset (GSE60361) was based on the cerebral cortex of a mouse and released by Zeisel et al. [12]. It included 3005 high-quality single cells containing unique molecular identifiers. These data were obtained via scRNA-seq technology (STRT/C1) for molecular reconnaissance of the hippocampus and somatosensory cortex of the cerebral cortex in mice. Each cell was represented by 19,972 genes.

The second dataset (GSE71585) was derived from the scRNA-seq data released by Tasic et al. [19]. It was used to construct the classification of primary visual cortex cells in adult mice (hereinafter referred to as visual cortex data). The data included seven types of cells: astrocytes, endothelial cells, GABA-ergic neurons, glutamatergic neurons, microglia, oligodendrocytes, and oligodendrocyte precursors. The details of the mouse visual cortex data are shown in Table 1.

Prabhakaran et al. [20] assumed that the gene expression vector *x_j_* of each cell after logarithmic phase was consistent with a Gaussian distribution and used the Lilliefors test to validate the assumption. Therefore, log_2_(*x*+1) was used to convert the single-cell expression data based on counts.

### 2.2. Measurements

The clustering accuracy (ACC) was adopted as the evaluation index. Let *c_i_* and *l_i_* be the cluster label and the label provided by the dataset, respectively. ACC is then defined as follows [21].

(1) $$ACC=\underset{m}{\max}\frac{\sum_{i=1}^{N}1\left\{l_{i}=m\left(c_{i}\right)\right\}}{n}$$

where *N* is the number of samples and *m*(*c_i_*) is the mapping function that maps each cluster label *c_i_* to the equivalent label from the dataset. Typically, the class label is provided by human experts. However, it is difficult to label single-cell RNA-seq data. GSE60361 and GSE71585 are the few labeled ones.

### 2.3. Visualization

*t*-distributed stochastic neighbor embedding (*t*-SNE) is a visualized nonlinear dimension reduction algorithm proposed by Maaten et al. [22]. This algorithm is based on stochastic neighbor embedding (SNE) and introduces *t*-distribution to reduce the crowding problem of SNE [23]. *t*-SNE uses the method of symmetric SNE to process high-dimensional data, whereas the sample data in the low-dimensional space adopts a symmetric *t*-distribution. The joint probability, *q_ij_*, which is the similarity between *y_i_* and *y_j_* in all samples in the low-dimensional space, is defined using a *t*-distribution of one degree of freedom:

(2) $$q_{ij}=\frac{{\left(1+{\left\|y_{i}-y_{j}\right\|}^{}\right)}^{-1}}{\sum_{k\neq j}{\left(1+\left\|y_{k}-y_{j}\right\|\right)}^{-1}}$$

(3) $$\frac{\delta C}{\delta y_{i}}=4\sum_{j}(p_{ij}-q_{ij})(y_{i}-y_{j}){\left(1+\left\|y_{i}-y_{j}\right\|\right)}^{-1}$$

where *p_ij_* is the similarity of two points in a high-dimensional space. *t*-SNE has been widely used in image processing, natural language processing, genomic data analysis, and speech processing [24,25,26].

### 2.4. Analyses of Mouse Cortex Data Results

Next, we analyzed the results according to the framework procedure. First, we discussed the necessary and effectiveness of feature selection (i.e., gene selection) and feature extraction on clustering. Subsequently, we analyzed the best combination of the dimensionality reduction and clustering algorithms using these datasets.

#### 2.4.1. Effectiveness of Feature Selection

The single-cell expression data contained many missing values and noise data, which affected the next step of the analysis (i.e., cell type identification). FBFS with variance was used to alleviate these problems. Inspired by Prabhakaran et al. [20], we selected the groups of genes with the largest expression variance. For the mouse cortex data, the original dimension was 19,972, and the number of cell samples was 3005. We adopted a feature gene-selection procedure to select genes with high variance. The variance represented the degree of differentiation the gene expression across all cells, and a high variance indicates that the gene was more important for distinguishing cells. Therefore, we could easily obtain the more biologically significant clusters.

**Figure 1:**
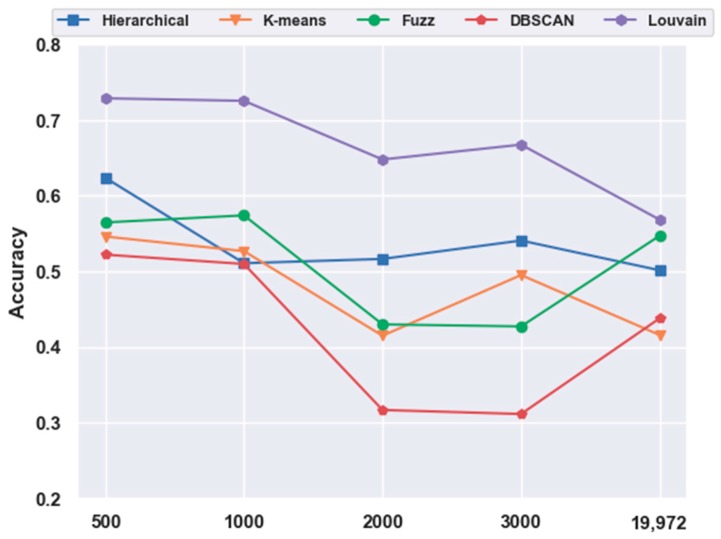
Effectiveness of feature selection on mouse cortex data. Hierarchical, K-means, Fuzz, DBSCAN, and Louvain represent hierarchical clustering, K-means, fuzzy C-means, density-based spatial clustering, and Louvain algorithms, respectively.

With FBFS, for the mouse cortex data, four subsets were generated with the top 500, 1000, 2000, and 3000 genes. We conducted the following experiments with five clustering models; the comparison results are shown in Figure 1. We compared all five clustering algorithms (i.e., hierarchical clustering, K-means, fuzzy C-means, DBSCAN, and Louvain) on the four subsets with the original data (19,972 genes with no feature extraction). The results of all five clustering models with the top 500 gene selection were better than those without feature selection (19,972 genes). Furthermore, the Louvain algorithm performed the best among these clustering algorithms, achieving an accuracy of 0.73 on 500 gene sets. The accuracy was 28.22% higher than the result without gene selection. Meanwhile, with the FBFS gene selection method, hierarchical clustering, K-means, fuzzy C-means, and DBSCAN achieved 24.34%, 31.3%, 3.22%, and 19.22% improvements in accuracy, respectively. These results showed that clustering with gene selection yielded a better performance compared with methods without it.

#### 2.4.2. Effectiveness of Feature Extraction

**Figure 2:**
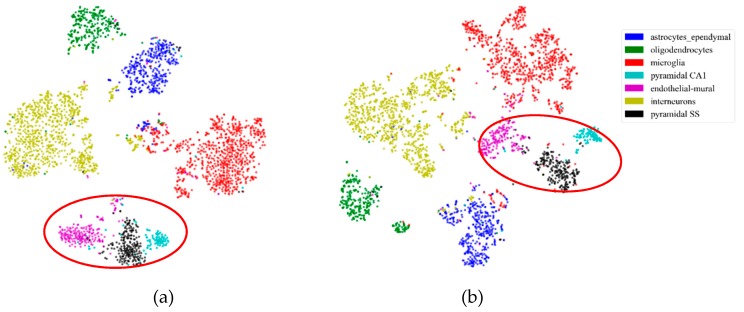
Comparison of no feature extraction (NFE) and non-negative matrix factorization (NMF) feature extraction. (**a**) The *t*-distributed stochastic neighbor embedding (*t*-SNE) graph of mouse cortex data of 500 genes with NFE. (**b**) The *t*-SNE graph of mouse cortex data of 500 genes with NMF. The red oval circled clustering results of the astrocyte ependymal cells, endothelial mural cells, and microglia cells.

First, to illustrate the effectiveness of feature extraction intuitively, we projected the mouse cortex data of 500 genes into a 20 dimensional feature space using the NMF feature-extraction model. The projection results are shown in Figure 2. From Figure 2a, we inferred that the astrocyte ependymal cells, endothelial mural cells, and microglia cells (in the red oval) were mixed together in the 500 gene space with no feature extraction (NFE). These three types of cells were classified into three groups using NMF feature extraction, as shown by the red oval in Figure 2b.

**Figure 3:**
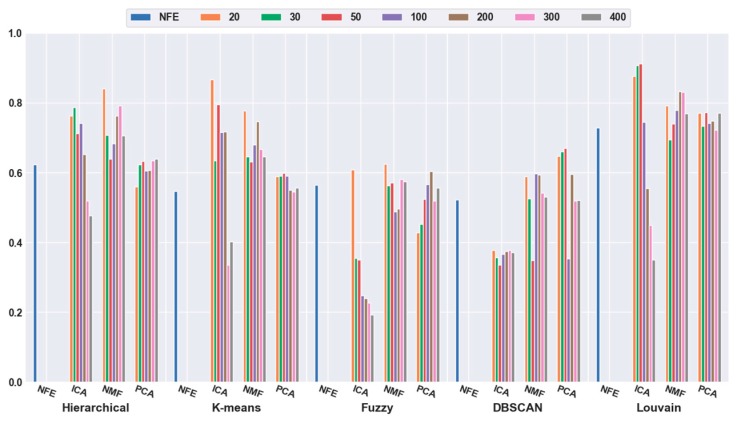
Comparison results of the five clustering methods with feature extraction on the mouse cortex data with 500 genes. From left to right, the blue, orange, green, red, brown, violet, khaki, pink, and gray bars indicate the results of five clustering models in the original feature space, 20 dimensional, 30 dimensional, 50 dimensional, 100 dimensional, 200 dimensional, 300 dimensional, and 400 dimensional feature spaces, respectively. ICA and PCA represent independent component analysis and principal component analysis algorithm.

To quantitatively validate the effectiveness of feature extraction, each clustering algorithm was combined with three dimensionality reduction algorithms. The number of dimensions was reduced to 20, 30, 50, 100, 200, 300, and 400. Figure 3 shows that the accuracies of all five clustering algorithms were significantly improved with the feature-extraction methods. For example, when the dimensions were reduced to 20 (orange bar), the combination of hierarchical clustering and NMF obtained a 34.69% higher accuracy than hierarchical clustering with NFE; the K-means-and-ICA-based combination model achieved a 58.18% higher accuracy than the K-means with NFE; the fuzzy C-means-and-NMF-based combination obtained a 10.60% higher accuracy than the NFE-based model; when the dimensions were reduced to 50 (red bar), the accuracy of the DBSCAN-and-PCA-based combination was 28.30% higher than that of DBSCAN with NFE; and the accuracy of the Louvain and ICA-based combination was 25.11% higher than that of Louvain with NFE. Based on all of the results described above, we concluded that a higher accuracy can be obtained using feature extraction.

Meanwhile, it was discovered that the selection of different feature-extraction models was crucial. For example, when the mouse cortex data with 500 genes was reduced to 50 dimensions by ICA, the highest clustering accuracy was achieved (accuracy = 0.93). This was achieved using Louvain clustering. Additionally, the second highest clustering result was 0.87, which was obtained using K-means in 20 dimensions.

**Figure 4:**
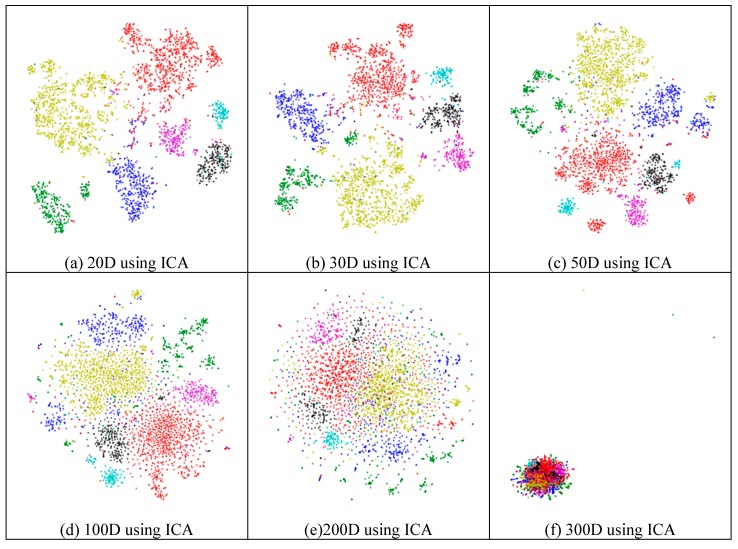

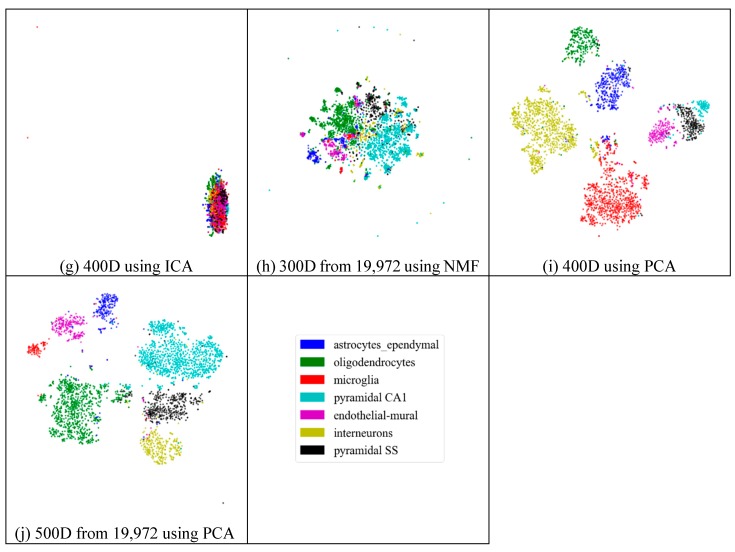
Comparison of different feature spaces and different extraction strategies on the mouse cortex data. (**a**) 20 dimensional feature space (20D) from 500 genes using ICA model. (**b**) 30 dimensional feature space (30D) from 500 genes using ICA model. (**c**) 50 dimensional feature space (50D) from 500 genes using the ICA method. (**d**) 100 dimensional feature space (100D) from 500 genes using the ICA model. (**e**) 200 dimensional feature space (200D) from 500 genes using the ICA model. (**f**) 300 dimensional feature space (300D) from 500 genes using the ICA model. (**g**) 400 dimensional feature space (400D) from 500 genes using the ICA model. (**h**) 300 dimensional feature space (300D) from 19,972 genes using the NMF model. (**i**) 400 dimensional feature space (400D) from 500 genes using the PCA model. (**j**) 500 dimensional feature space (500D) from 19,972 genes using the PCA model.

Next, we assessed whether feature-extraction methods always benefit scRNA-seq data. To validate the benefit of feature extraction, we compared the clustering results for different scales of feature space and different extraction strategies. As shown in Figure 4, ICA extracted the features from 500 genes to 20 features (Figure 4a), 30 features (Figure 4b), 50 features (Figure 4c), 100 features (Figure 4d), 200 features (Figure 4e), 300 features (Figure 4f), and 400 features (Figure 4g), respectively. From Figure 4a–c, it is clear that all seven types of cells could be distinguished; however, in the following four subfigures (Figure 4d–g), different cells were mixed and difficult to separate. This means that in these four generated feature spaces, satisfactory results were difficult to achieve.

In Figure 4h (300 features extracted from 19,972 genes by NMF), the results exhibited disordered groups. This was similar to the ICA results with 300 features (Figure 4f). This means that NMF and ICA could not always achieve good results, especially for larger feature spaces. However, the PCA graphs did not show apparent disorder for either the feature-selected data (Figure 4i) or the original data (Figure 4j). This indicates that the PCA strategy was more robust than the other feature-extraction methods.

#### 2.4.3. Which Clustering Algorithm Is Better?

**Figure 5:**
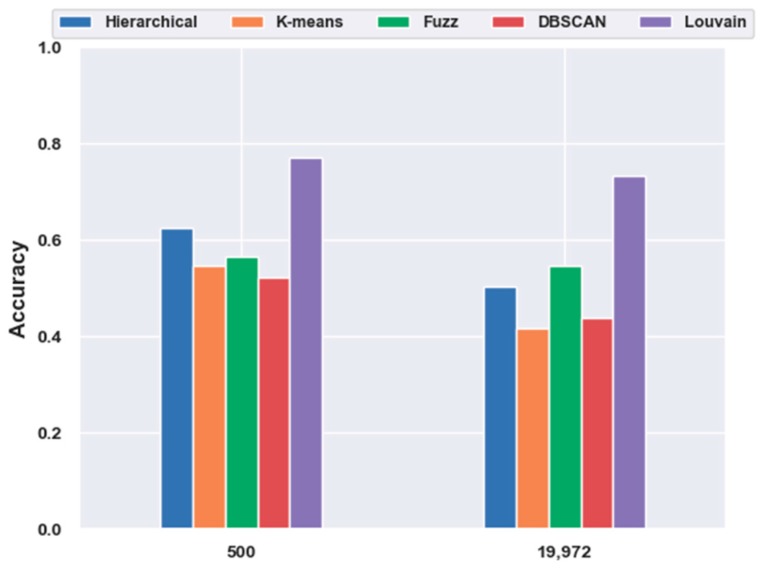
Comparison of the five clustering models with NFE on the mouse cortex data. The results of 19,972 genes are from the original data without dimensionality reduction. The results of 500 genes are from the mouse cortex data of 500 genes with feature selection.

To investigate the effectiveness of clustering models without feature-extraction models, we directly performed clustering on the original data and feature-selection data for 500 genes. The results are illustrated in Figure 5. We discovered that without any feature-extraction method, Louvain clustering achieved the best results on the original and feature-selected data. On the original data, Louvain reached an accuracy of 0.73, which was 46.18%, 76.24%, 34.00%, and 67.40% higher than those of hierarchical clustering, K-means, fuzzy C-means, and DBSCAN, respectively. For the 500 genes, Louvain achieved an accuracy of 0.77, which was 23.43%, 40.95%, 36.30%, and 47.42% higher than those of the other four clustering models, respectively. Moreover, with feature selection, the clustering accuracy yielded a 5.48% increase from 0.73 to 0.77. This coincided with the aforementioned conclusion regarding feature selection.

In addition, the parameter *k* of K-means was set as the number of cell types. The *k* of k-nearest neighbor algorithm (kNN) used in the Louvain model was set based on {20, 50, 60, 70, 80, 100, 120, 150}. When *k* = 70 in 500 gene subset and *k* = 20 in original dataset, Louvain obtained the best results, as shown in Figure 5. Additionally, all of the Louvain results presented in Figure 3 were achieved when *k* = 80.

#### 2.4.4. Which Combination Is Better?

**Figure 6:**
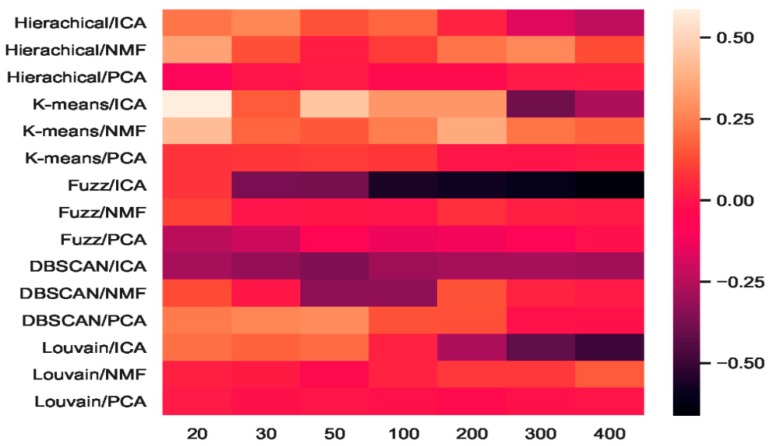
Heatmap of clustering accuracy improvement using feature extraction on the mouse cortex with 500 genes.

We combined three feature-extraction models (i.e., ICA, NMF, and PCA) with five clustering models (hierarchical clustering, K-means, fuzzy C-means, DBSCAN, and Louvain), and 15 combinations were generated. The baselines were direct clustering results without combination. In Figure 6, the heatmap of accuracy improvement for the combinations in the mouse cortex with the 500 gene set is shown. Over half of the clustering results in red demonstrated a positive effect being achieved, whereas the negative results are shown in black. The accuracy of ICA + K-means increased from 0.55 to 0.87 and achieved the highest increase of 58.18%, as shown by the brightest red color in Figure 6. However, when dimensions were greater than 200, the accuracy of K-means + ICA became worse than the baseline and is represented by darker color.

From the results shown in Figure 5, we inferred that Louvain is the best clustering method for this dataset. As shown in Figure 6, the combination of Louvain and ICA achieved red promotion when the feature space was less than 100 dimensions. This result means that ICA further improved the Louvain clustering accuracy in small feature spaces. The combination of Louvain + ICA achieved the highest accuracy (ca. 0.93, 0.92, and 0.91) on this dataset. However, when the number of dimensions increased, the Louvain and ICA combination results worsened. For example, on dimensions of 200, 300, and 400, Louvain + NMF achieved red promotion (24.45%, 24.15%, and 15.09% higher than the baseline), whereas Louvain + ICA showed a dark decline (31.77%, 68.25%, and 79.40% lower than the baseline). In these feature spaces, the former combination achieved better results, which were 50.06%, 85.01%, and 119.56% higher than those of the latter. From the *t*-SNE graphs of Figure 4e–g, it is clear that the cells in these new projected feature spaces were mixed together. Therefore, Louvain + ICA could not perform well on these feature spaces. Furthermore, the combinations did not always benefit scRNA-seq data clustering.

Although Louvain + ICA achieved the best clustering result and K-means + ICA achieved the highest red promotion, the combinations of fuzzy C-means + ICA and DBSCAN + ICA did not perform as well as the former two combinations. As shown in Figure 6, DBSCAN + ICA indicated a dark decline. This means that this combination yielded worse results in all seven extracted feature spaces relative to the baseline of DBSCAN. The accuracy-improvement pattern of Hierarchical + ICA was similar to that of K-means + ICA, which achieved red promotion with less than 200 dimensions.

Compared with the high fluctuation of the ICA-based combinations, we discovered that the other eight combinations of hierarchical + PCA, K-means + PCA, DBSCAN + PCA, Louvain + PCA, hierarchical+ NMF, K-means + NMF, fuzzy C-means + NMF, and Louvain + NMF all achieved red promotion (Figure 6). Fuzzy C-means + PCA underwent a dark decline at the beginning (e.g., 20 dimensions and 30 dimensions), whereas DBSCAN + NMF was dark in the middle (50 and 100 dimensions). Considering the robustness of the combinations, PCA + NMF did not fluctuate significantly with dimension changes, and the performance was relatively stable.

### 2.5. Analyses of Mouse Visual Cortex Data Results

To determine the universality of the aforementioned discoveries, we performed experiments on mouse visual cortex data. After comparing the mouse cortex data, we presented the similarities and differences between the results.

#### 2.5.1. Consistency Results

**Figure 7:**
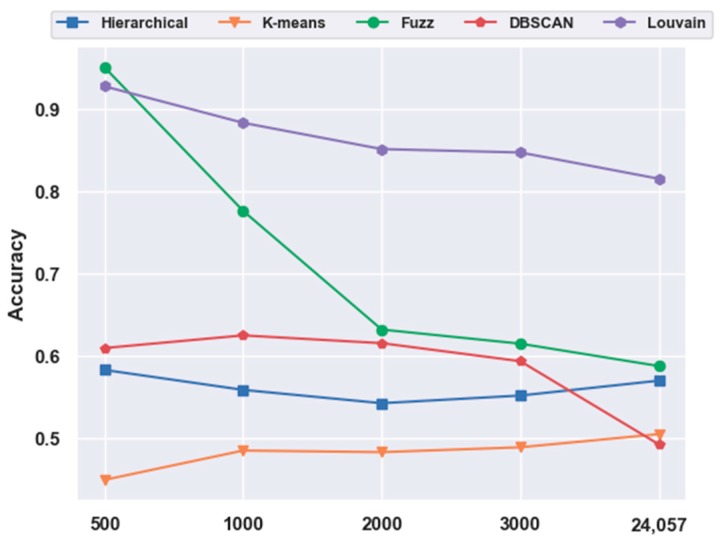
Effectiveness of gene selection on mouse visual cortex data.

For the mouse cortex data, gene selection helped to achieve better performances. With FBFS, for the visual mouse cortex data, four subsets with the top 500, 1000, 2000, and 3000 genes were generated. We compared five clustering models on 500 gene feature selection data with no feature-extraction data (24,057 genes). As shown in Figure 7, most of the results (four in five, except K-means) with gene selection were better than those without gene selection. The four algorithms, hierarchical clustering, fuzzy C-means, DBSCAN, and Louvain, achieved 16.96%, 61.83%, 23.80%, and 13.79% improvements in accuracy, respectively.

**Figure 8:**
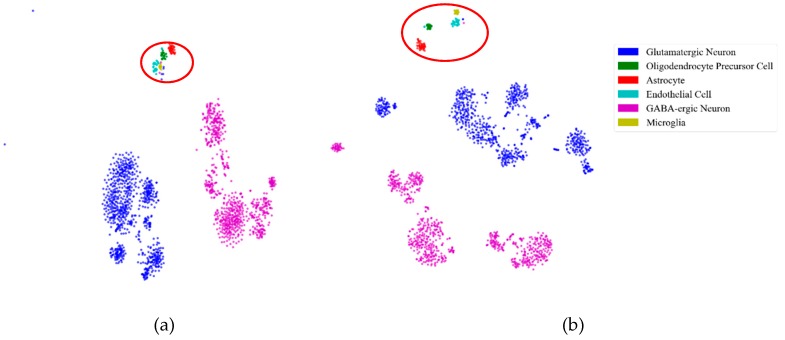
Effectiveness of feature extraction on mouse visual cortex data. (**a**) The *t*-SNE graph of mouse visual cortex data of 500 genes with NFE. (**b**) The *t*-SNE graph of the mouse visual cortex data of 500 genes with PCA. The read oval circled different clustering results of 500 genes with NFE and PCA.

Similarly to the mouse cortex data, Figure 8 illustrates the effectiveness of feature extraction, as shown in Figure 2. We projected the mouse visual cortex data of 500 genes into a 50 dimensional feature space using the PCA feature-extraction model. From Figure 8a, we inferred that the astrocytes, oligodendrocyte precursor cells, endothelial cells, and microglia (in the red oval) were mixed together in the 500 gene space. With PCA feature extraction, these three types of cells could be categorized into four groups, as shown in the red oval in Figure 8b.

**Figure 9:**
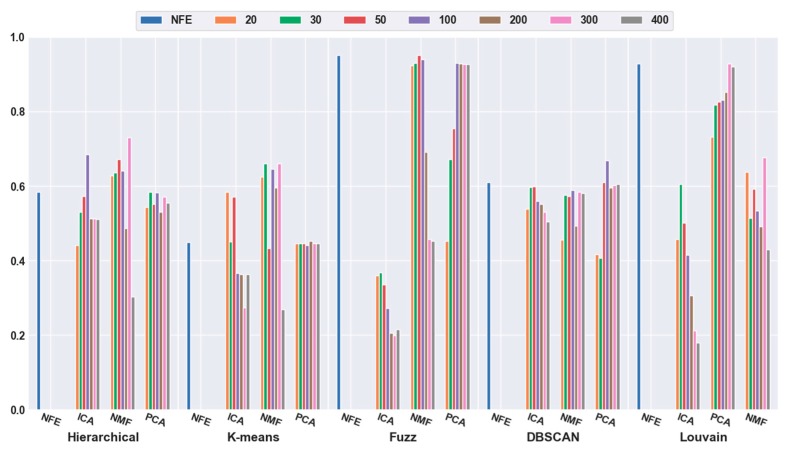
Comparison of the five clustering models with feature extraction on the mouse visual cortex data with 500 genes. From left to right, the blue, orange, green, red, brown, pink, gray, and khaki bars indicate the results of five clustering models in the original feature space and the 20 dimensional, 30 dimensional, 50 dimensional, 100 dimensional, 200 dimensional, 300 dimensional, and 400 dimensional feature spaces, respectively.

Moreover, regarding the effectiveness of feature extraction, from Figure 9, we investigated whether the accuracies of three clustering algorithms (hierarchical clustering, K-means, and DBSCAN) can be improved with the feature-extraction models. For example, when the dimensions were reduced to 300 (pink bar), the hierarchical-clustering-and-NMF-based combination obtained a 25.15% higher accuracy than hierarchical clustering with NFE; the K-means-and-NMF-based combination achieved a 46.77% higher accuracy than K-means with NFE. When the dimensions were reduced to 100 (the violet bar), the accuracy of the DBSCAN-and-PCA-based combination was 9.71% higher than that of DBSCAN with NFE. The fuzzy C-means and Louvain models with feature-extraction model achieved the same accuracy as that of the model with NFE. Based on these results for the mouse visual cortex data with 500 genes, we concluded that feature extraction enabled the clustering models to achieve better performance in most cases (three out of five achieved better performance, whereas the other two achieved equal performance).

**Figure 10:**
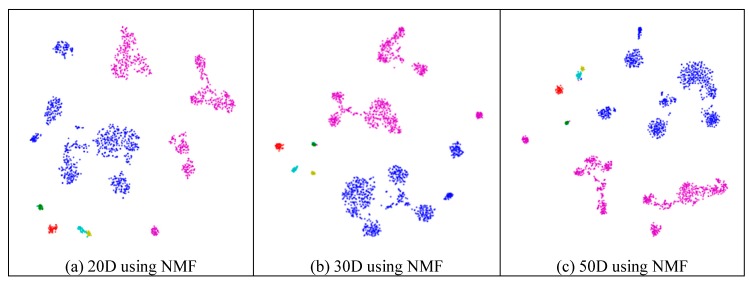

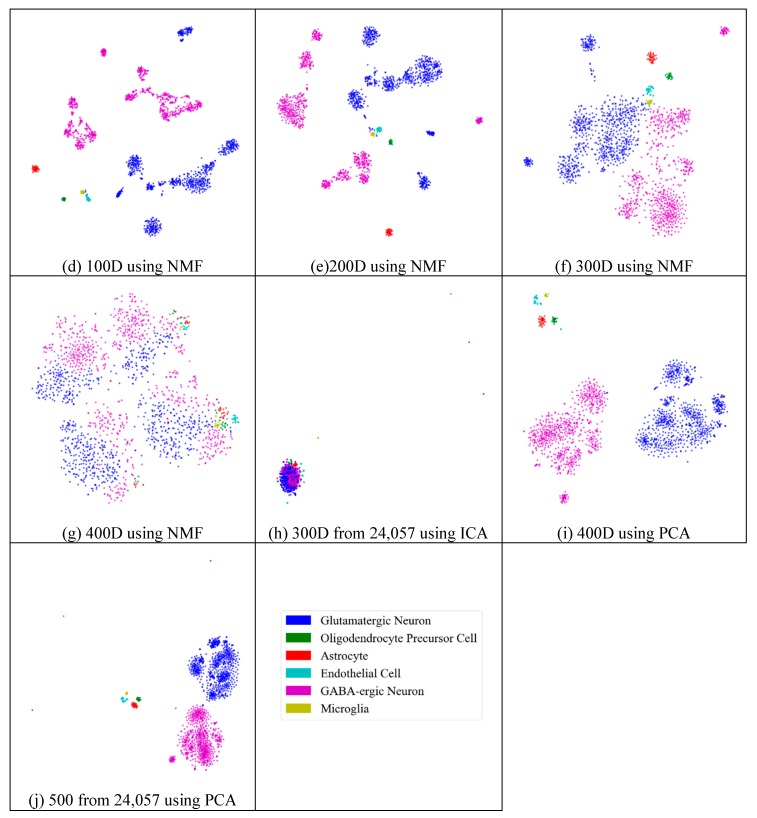
Comparison of different feature spaces and different extraction strategies on the mouse visual cortex data. (**a**) 20 dimensional feature space (20D) from 500 genes using the NMF model. (**b**) 30 dimensional feature space (30D) from 500 genes using the NMF model. (**c**) 50 dimensional feature space (50D) from 500 genes using the NMF model. (**d**) 100 dimensional feature space (100D) from 500 genes using the NMF model. (**e**) 200 dimensional feature space (200D) from 500 genes using the NMF model. (**f**) 300 dimensional feature space (300D) from 500 genes using the NMF model. (**g**) 400 dimensional feature space (400D) from 500 genes using the NMF model. (**h**) 300 dimensional feature space (300D) from 24,057 genes using the ICA model. (**i**) 400 dimensional feature space (400D) from 500 genes using the PCA model. (**j**) 500 dimensional feature space (500D) from 24,057 genes using the PCA model.

Similarly to the results from the mouse cortex data, feature extraction did not always improve the clustering performance. As shown in Figure 10, we compared the results of different feature spaces and different feature-extraction models on the mouse visual cortex data. NMF extracted the feature space from 500 genes to 20 features (Figure 10a), 30 features (Figure 10b), 50 features (Figure 10c), 100 features (Figure 10d), 200 features (Figure 10e), 300 features (Figure 10f), and 400 features (Figure 10g). From Figure 10a–e, all six types of cell could be distinguished. However, in the following two subfigures (Figure 10f,g), these cells were mixed and difficult to classify. Therefore, in the two feature spaces, the clustering algorithms could not achieve satisfactory results. For the ICA model, Figure 10h (300 features extracted from 24,057 genes by ICA) showed a disordered group; however, PCA did not show apparent disorder in either the feature-selected data (Figure 10i) or the original data (Figure 10j). Similarly to the results from the former data, PCA performed better than the other feature-extraction methods.

#### 2.5.2. Different Results

**Figure 11:**
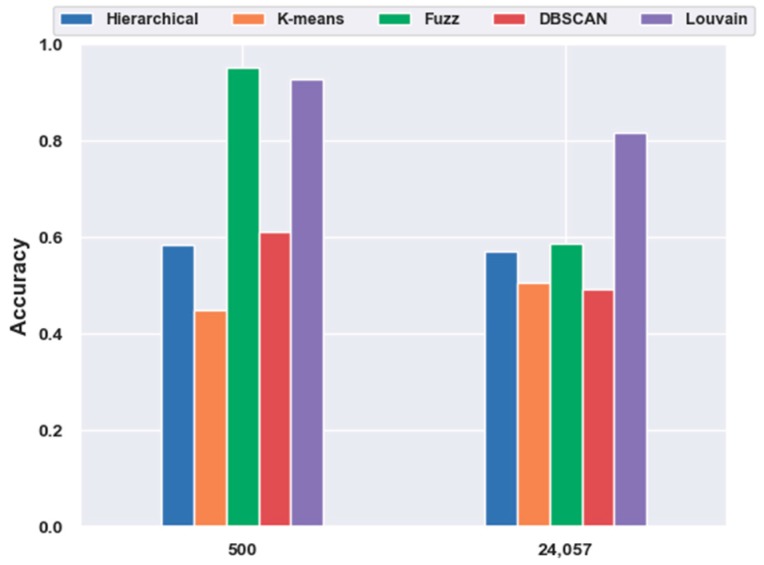
Comparison of the five clustering models with NFE on the mouse visual cortex data. The results from 24,057 genes were derived from the original data without dimensionality reduction. The results of 500 genes were derived from the mouse cortex data of 500 genes with feature selection. Additionally, the *k* values of k-nearest neighbor algorithm (kNN) used in the Louvain models were set to 500 in the 500 gene subset and 200 in the original dataset.

In contrast to Figure 1, in Figure 7, fuzzy C-means instead of the Louvain model performed the best on the 500 gene subset, achieving an accuracy of 0.95. As shown in Figure 11, unlike in Figure 5, without the feature-extraction model, fuzzy C-means achieved the best results on the feature-selected data, whereas Louvain performed the best on the original data. On the original data, Louvain achieved an accuracy of 0.69, which was 12.80%, 42.58%, 18.02%, and 40.87% higher than those achieved with hierarchical clustering, K-means, fuzzy C-means, and DBSCAN, respectively. On 500 gene feature selected data, fuzzy C-means achieved an accuracy of 0.95, which was 62.98%, 117.73%, 56.02%, and 2.49% higher than those of the other four clustering models, respectively. However, as shown in Figure 5, Louvain was the best clustering model without feature extraction on the mouse cortex data.

**Figure 12:**
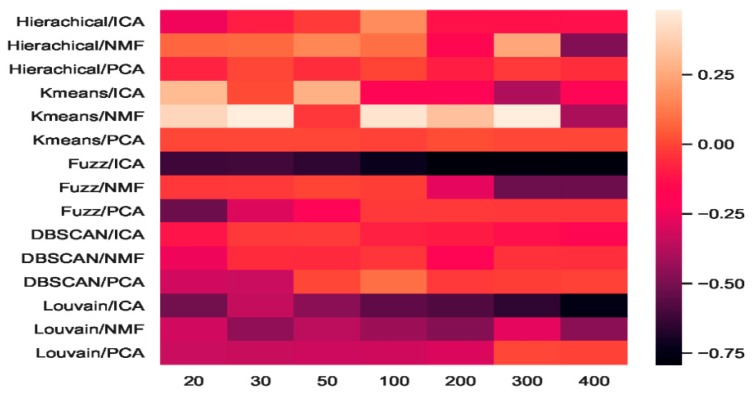
Heatmap of clustering accuracy improvement using feature extraction on 500 genes of the mouse visual cortex.

The heatmap of accuracy improvement on the visual mouse cortex data (Figure 12) illustrates that the combinations of feature-extraction and clustering models did not perform as well as on the mouse cortex data. As shown in Figure 12, 24.82% of the clustering results (in red) achieved a positive effect, whereas the negative results are shown in black. The accuracy of NMF + K-means increased from 0.45 to 0.66, achieving the highest increase of 46.67%, represented by the brightest red in Figure 12. However, when the number of dimensions was larger than 300, the accuracy of K-means + NMF became worse than that of the baseline, and is represented by a darker color. On the mouse cortex data, K-means + ICA achieved the highest promotion (see Figure 6).

As shown in Figure 12, the results of fuzzy C-means + ICA declined, as represented by darker colors. This means that this combination performed worse in all seven extracted feature spaces than the fuzzy C-means baseline. However, this combination achieved red promotion in the 20 feature space shown in Figure 6. Compared with the eight combinations that achieved red promotion in all feature spaces in Figure 6, Figure 12 only shows three combination (i.e., hierarchical + PCA, K-means + PCA, and DBSCAN + ICA).

## 3. Discussion

Based on mouse cortex and mouse visual cortex data, four conclusions were drawn from our experiments. First, in addressing the problem of high-dimensional and sparse matrix computation, feature selection contributed positively to scRNA-seq data analyses. This is because feature selection is one of the simplest but most effective methods to reduce the effect of the curse of dimensionality. When the feature space was limited to the selected features, the gene-expression matrix became dense, which eased clustering.

Second, feature extraction enhanced the clustering performance on the scRNA-seq data; however, this was not eternally immutable. For the three typically used feature-extraction models, ICA performed the best on the mouse cortex data, whereas PCA was steadier. ICA achieved good results in small feature spaces, but its performance deteriorated when the number of features increased. This was because some of the expressed genes were dependent for the scRNA-seq data. Many genes exhibited strong relatedness with each other and were expressed together for biological functions. Therefore, we could project them into independent components.

Third, for the five clustering models, Louvain performed better than the other four clustering algorithms on the first dataset. For the second dataset, Louvain ranked second, whereas fuzzy C-means clustering performed the best. 

Fourth, when using K-means clustering, feature-extraction models should be adopted to achieve good results. This is because in high-dimensional and sparse feature spaces, K-means clustering falls into local optimization more easily. ICA is not suitable for fuzzy C-means clustering because of the poor performance of this combination on our two scRNA-seq datasets.

## 4. Materials and Methods

### 4.1. Dimensionality Reduction Models

Dimensionality reduction refers to reduction in the number of features, and its approaches can be categorized into feature selection and feature extraction [23,27]. Feature selection models can be classified into three main categories: wrappers, filters, and embedded methods [28,29]. Because filtered features can achieve a more general result and are not tuned to a specific type of predictive model, we focused on the FBFS model. Moreover, FBFS is typically less computationally intensive than wrappers and can be constructed with prior knowledge; however, this knowledge is difficult to obtain. We selected the groups of features (i.e., genes, in our experiments) with the largest expression variance and ranked them for further analysis.

PCA is a linear feature-extraction algorithm that is widely used in biological research [30]. The main idea of PCA is to project the feature space from high to low dimensions and reconstruct the *k*-dimensional orthogonal features from the original *n*-dimensional feature space. PCA is an important tool for analyzing high-dimensional gene expression data and has been used with scRNA-seq data [31].

ICA [32] can be used to obtain hidden factors from multidimensional data. The goal of ICA is to decompose a multivariate signal into independent non-Gaussian components such that the components are statistically independent, or as independent as possible. ICA assumes observation *X* as a linear mixture of independent components *S*. Let *A* denote the inverse matrix of the weight matrix *W*, and the columns of *A* represent the basis feature vectors of observation *X*.

*S* = *W* × *X*, *X* = *A* × *S* (4)

ICA has been widely used in blind source separation, image processing, speech recognition, biological information, and other fields [33,34,35,36].

NMF [37] is a matrix decomposition algorithm and can be described as follows. Given a non-negative matrix *V* ∈ *R^N^*^×*M*^ and constant rank *k,* NMF finds a non-negative *n* × *k* matrix *W* and another non-negative *k* × *m* matrix *H*. Additionally, *W* × *H* approximates to *V, V* *≈ W* × *H,* where *k* is significantly less than *M* and *N*. NMF is applicable to many fields, such as image feature recognition [38], speech recognition [39], biomedical engineering [40,41], and document clustering [21,42].

### 4.2. Clustering Models

Most clustering models can be classified into five different categories: hierarchical clustering, squared error-based clustering, graph theory-based clustering, fuzzy clustering, and density-based spatial clustering algorithms. We selected one from each category and investigated these classical and widely used clustering algorithms.

Hierarchical clustering [13] is a structured clustering algorithm that segregates data into different levels to achieve a tree structure based on the similarity between data points of different categories. In this tree, the original data point is the leaf of the tree, and the top of the tree is the root node. Generally, two types of strategies exist for hierarchical clustering: agglomerative (bottom-up approach) and divisive (top-down approach). We selected the former in our experiment because of its wider application.

The K-means algorithm is a simple and fast squared error-based clustering method that has been applied in many fields [43,44]. Its basic idea is as follows. Given a set of data, (*x*_1_, *x*_2_, *…, x_n_*), each of these points is a real vector in *d* dimensions, and the purpose of K-means is to group *n* points into *k*(≤ *n*) clusters, *s = (s*_1_*, s*_2_*, …, s_k_)* to yield the minimum variances in each cluster. Generally, with the Euclidean distance, the goal is to obtain

(5) $$\arg\underset{S}{\min}\sum_{i=1}^{k}{\sum_{x\in S_{i}}\left\|x-\mu_{i}\right\|}^{2}=\arg\underset{s}{\min}\sum_{i=1}^{k}\left|S_{i}\right|Var(S_{i})$$

where *μ_i_* is the mean of the points in *s_i_*. K-means is a greedy algorithm to minimize the squared deviation of the data points in the same cluster:

(6) $$\arg\underset{S}{\min}\sum_{i=1}^{k}\frac{1}{2\left|S_{i}\right|}{\sum_{x,y\in S_{i}}\left\|x-y\right\|}^{2}$$

Fuzzy C-means is a classical fuzzy clustering algorithm proposed by Dunn and Bezdek [45]. It minimizes the objective function to obtain the membership degree of each sample to each cluster center. The objective function is shown in Equation (7).

(7) $$J_{m}=\sum_{i=1}^{N}\sum_{j=1}^{c}u_{ij}^{m}\|x_{i}-c_{j}\|{}^{2},\;1\leq m<\infty$$

This objective function is minimized through the update iteration of the following two equations.

(8) $$u_{ij}=\frac{1}{\sum_{k=1}^{c}{\left(\frac{\left\|x_{i}-c_{j}\right\|}{\left\|x_{j}-c_{k}\right\|}\right)}^{\frac{2}{m-1}}}$$

(9) $$c_{j}=\frac{\sum_{i=1}^{N}u_{ij}^{m}x_{i}}{\sum_{i=1}^{N}u_{ij}^{m}}$$

DBSCAN is a density-based clustering algorithm that can be used to solve the clustering problem of irregular shapes. It uses the high-density connectivity of the clusters and seeks a high-density area that is separated by a low-density area. DBSCAN can discover clusters of arbitrary shapes. For each sample in a cluster, the number of objects must exceed the given minimum number in its domain of a given radius. Ester et al. [46] evaluated the effectiveness and efficiency of DBSCAN using synthetic data and real data from the SEQUOIA 2000 benchmark, and proved that DBSCAN performed more effectively at discovering clusters of arbitrary shapes than the CLARANS(Clustering Large Applications based on Randomized Search) algorithm.

The Louvain method for community detection is a heuristic method based on modularity optimization [17]. It belongs to graph-theory-based clustering, and its advantages are its high accuracy and efficiency. It is considered as one of the best algorithms in community discovery [47]. The Louvain method is a multistep technique based on the local optimization of the Newman–Girvan modularity for each node. The modularity function is used to compute the compactness of the community and achieve the optimization objective of the algorithm. This maximizes the modularity *Q* of the entire sample set. *Q* is calculated as follows:

(10) $$Q=\frac{1}{2m}\sum_{i,j=0}^{n}\left[A_{ij}-\frac{k_{i}k_{j}}{2m}\right]\delta (c_{i},c_{j})$$

where *m* is the number of edges in the network; *k_i_* and *k_j_* represent the sum of all edge weights pointing to nodes *i* and *j*, respectively; *A_ij_* represents the edge weight between nodes *i* and *j*; *c_i_* represents the community where node *i* is located, and each community is a cluster; and *δ(c_i_,c_j_)* is obtained using Equation (11).

(11) $$\delta (c_{i},c_{j})=\left\{ \begin{array}{ll} 1 & c_{i}=c_{j}\\ 0 & \mathrm{otherwise} \end{array}\right.$$

This method offers a compromise between the accuracy of the estimate of modularity maximum and the computational complexity, which is linear with the number of links in the graph.

### 4.3. Comparative Framework

**Figure 13:**
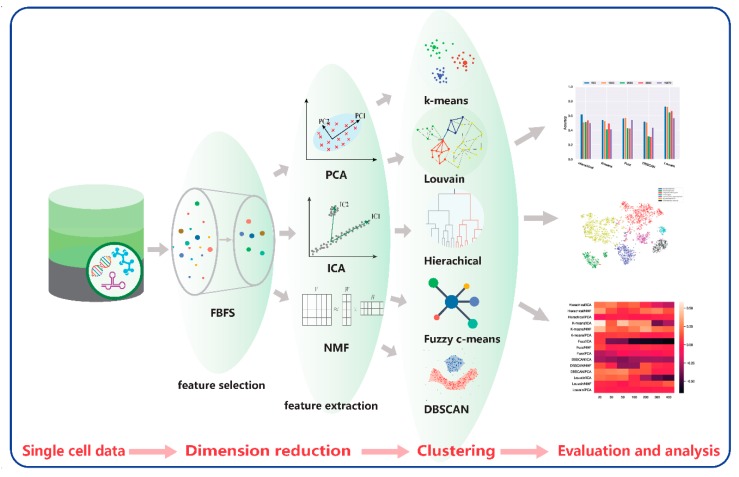
Comparative framework.

Dimensionality reduction and clustering are important in scRNA-seq data analysis. We propose a framework that combines four dimension-reduction models and five clustering models. Comparison results are provided below based on these combinations for two scRNA-seq datasets to illustrate the effectiveness of these models. The framework is shown in Figure 13.

Our proposed framework contains four modules. The first module is data acquisition and preprocessing. The second module is the dimensionality reduction module, including FBFS, PCA, NMF, and ICA for highly dimensional scRNA-seq data. Subsequently, for the clustering module, we adopted hierarchical clustering, K-means, fuzzy C-means, DBSCAN, and Louvain. The last module is the visualization and evaluation. We performed a quantitative analysis of the data results and a visual analysis using *t*-SNE. The analysis included the performance of each clustering algorithm on the scRNA-seq data and the effect of dimensionality reduction on clustering. The details of the 20 combinations are listed in Table 2.

To introduce the Louvain algorithm into scRNA-seq data analysis, a nearest neighbor graph was constructed using the kNN. For each cell sample, the Euclidean distance was used to compute the similarity between the current cell and other cells. The nearest *k* samples were used to construct the adjacency graph *A*. For sample *i*, *A_ij_* = 1 if sample *j* is one of the *k* samples closest to sample *i*; otherwise, *A_ij_* = 0. When building a single-cell neighbor graph, the number of neighbors must be selected, which will affect the number and size of the final cluster. Therefore, selecting a suitable *k* is crucial, and is discussed in the results section.

## 5. Conclusions

Dimensionality reduction and clustering are important in scRNA-seq data analysis. A comparative framework is proposed which combines four dimension-reduction models and five clustering models. Four experiments were progressively performed on two large scRNA-seq datasets using these combinations. Four conclusions are drawn from the results. In summary, feature selection is crucial for achieving better clustering results. When the result is unsatisfactory, feature-extraction methods can be introduced, especially for K-means, hierarchical clustering, and DBSCAN. Louvain clustering can obtain satisfactory results in most cases.

## Figures and Tables

**Table 1:** Cell type and number of mouse visual cortex data.

Broad Type	Count
Astrocyte	43
Endothelial Cell	29
GABA-ergic Neuron	761
Glutamatergic Neuron	812
Microglia	22
Oligodendrocyte	38
Oligodendrocyte Precursor	22
Unclassified	82

**Table 2:** Combinations of dimensionality reduction and clustering models.

Combination Numbers	Combination Mode	Field
1	K-Means	mouse retinal cells [48] peripheral blood mononuclear cell [49]
2	Hierarchical Clustering	intestinal cell types [50] adult brain cell [51] embryonic mouse lung [52] human preimplantation embryos and embryonic stem cells [53]
3	Louvain	progenitor-like cells [54] peripheral blood mononuclear cell [18]
4	Fuzzy C-Means	rare intestinal cell type in mice [55] Genotype-tissue Expression (GTEx) human tissue dataset [56]
5	DBSCAN ^1^	B-cell Lymphoma [57]
6	PCA ^2^ + K-Means	intestinal cell types [50] Lung epithelial cells [58]
7	PCA + Hierarchical Clustering	human and mouse early embryo [59] distal lung epithelium [60] breast-cancer-associated endothelial cells [61]
8	PCA + Louvain	retinal bipolar neurons [62]
9	PCA + Fuzzy C-Means	rare intestinal cell type in mice [55]
10	PCA + DBSCAN	mouse retinal cells [63]
11	NMF ^3^ + K-Means	renal cell carcinoma, liver cancer, lung cancer [64]
12	NMF + Hierarchical Clustering	mouse strain [65]
13	NMF + Louvain	unreported model
14	NMF + Fuzzy C-Means	unreported model
15	NMF + DBSCAN	unreported model
16	ICA ^4^ + K-Means	individual cell [66] adult hippocampal quiescent neural stem cell [67]
17	ICA + Hierarchical Clustering	Physcomitrella leaf cell [68]
18	ICA + Louvain	human aging lung [69]
19	ICA + Fuzzy C-Means	unreported model
20	ICA + DBSCAN	unreported model

^1^ DBSCAN represents density-based spatial clustering of applications with noise.

^2^ PCA represents principal component analysis.

^3^ NMF represents non-negative matrix factorization.

^4^ ICA represents independent component analysis.
